# The Influence of the Specimen Shape and Loading Conditions on the Parameter Identification of a Viscoelastic Brain Model

**DOI:** 10.1155/2013/460413

**Published:** 2013-07-09

**Authors:** Costin D. Untaroiu

**Affiliations:** Virginia Tech & Wake Forest School of Biomedical Engineering and Sciences, Center for Injury Biomechanics, 2280 Kraft Drive, Blacksburg, VA 24060, USA

## Abstract

The mechanical properties of brain under various loadings have been reported in the literature over the past 50 years. Step-and-hold tests have often been employed to characterize viscoelastic and nonlinear behavior of brain under high-rate shear deformation; however, the identification of brain material parameters is typically performed by neglecting the initial strain ramp and/or by assuming a uniform strain distribution in the brain samples. Using finite element (FE) simulations of shear tests, this study shows that these simplifications have a significant effect on the identified material properties in the case of cylindrical human brain specimens. Material models optimized using only the stress relaxation curve under predict the shear force during the strain ramp, mainly due to lower values of their instantaneous shear moduli. Similarly, material models optimized using an analytical approach, which assumes a uniform strain distribution, under predict peak shear forces in FE simulations. Reducing the specimen height showed to improve the model prediction, but no improvements were observed for cubic samples with heights similar to cylindrical samples. Models optimized using FE simulations show the closest response to the test data, so a FE-based optimization approach is recommended in future parameter identification studies of brain.

## 1. Introduction

Traumatic Brain Injury (TBI) has severe consequences and can be life threatening. According to the Center for Diseases and Control and Prevention, TBI is an important public health problem in the United States affecting approximately 1.7 million people and causing 52,000 deaths each year [1]. While falls are the leading cause of TBI, the motor vehicle-traffic accidents are the leading cause of TBI-related death [1]. 

The continuous improvement of computational human models [2] and optimization techniques [3] may help in the development of brain injury countermeasures [4, 5]; however, valid brain numerical models require accurate material models under a variety of loading conditions.

The mechanical properties of brain under various loadings have been reported in many studies in the literature over the past 50 years [6]. Step-and-hold tests under simple shear loading are often used to characterize viscoelastic and nonlinear behavior of brain under high-rate deformation [6–10]. However, the stress relaxation curves used for material identification of brain are typically obtained under two assumptions. First, by neglecting the initial strain ramp, a perfect step loading is assumed [11]. Second, a uniform strain distribution is assumed, and the parameters of a one-dimensional (1D) analytical material model are identified using optimization techniques [9].

In an effort to better understand the effects of the aforementioned two assumptions on the identified material properties of brain, a finite element (FE) approach, which considers the three-dimensional (3D) deformation of the sample, is employed in this study.

## 2. Materials and Methods

### 2.1. Shear Testing

Test data were taken from simple shear tests of seven cylindrical human samples collected by Takhounts [11]. Fresh human brain samples (approximately 12 mm height and 18 mm diameter) of primarily white matter (more than 85% according to histological analysis of samples) were obtained within 24 hours of death and were kept moist and refrigerated during the next 24 hours. The samples were attached to the plates of a custom-made testing device using methyl-2-cyanoacrylate adhesive (Super Glue Corporation, Rancho Cucamonga, CA) [11]. A programmable linear actuator attached to the lower plate was used to apply a linear displacement to the brain sample corresponding to 50% engineering shear strain in about 0.1 sec. Two force transducers (Sensotec Inc., Columbus, Ohio, Model 31/1435-03-04 and Model 31/1434-01-01) attached to the upper (fixed) plate were used to record the shear force during the testing.

### 2.2. Material Identification: Analytical Approach

It is well known that brain tissue exhibits time-dependent stress-strain behavior [12]. The *isotropic linear viscoelasticity* (LV) is one of the simplest constitutive formulations used to model the brain. Compared to other more complex formulations, such as quasi-linear viscoelasticity [7–10, 13] and nonlinear viscoelasticity [12, 14, 15], LV models are easy to be assigned because they are implemented in the majority of FE software packages. According to LV formulation, if the material is subjected to a perfect strain step (*ε*_0_):

(1) $$\begin{array}{c} \varepsilon \left(t\right)=\varepsilon_{0}H\left(t-\tau \right), \end{array}$$

where *H* is Heaviside step function, then the stress is given by

(2) $$\begin{array}{c} \sigma \left(t\right)=\varepsilon_{0}G\left(t\right), \end{array}$$

where *G*(*t*) is the stress relaxation function which is often approximated as a sum of exponentials

(3) $$\begin{array}{c} G\left(t\right)=G_{\infty }+\sum_{i=1}^{3}G_{i}^{-\beta_{i}t}. \end{array}$$

*G*_*∞*_, *G*_0_ = *G*_*∞*_ + ∑_*i*=1_^3^ *G*_*i*_, and *G*_*r*_(*t*) = *G*(*t*)/*G*_*∞*_ are the long term shear modulus, the instantaneous shear modulus, and the reduced shear relaxation, respectively. Applying the Boltzmann superposition principle, the stress time history has the following integral representation:

(4) $$\begin{array}{c} \sigma \left(t\right)=\int_{0}^{t}G\left(t-\tau \right)\frac{\partial \sigma \left(\varepsilon \right)}{\partial \tau }d\tau . \end{array}$$

In testing, the ramp time was about 0.1 sec and the total duration of the ramp-and-hold test was about 4 sec, so three decay rates with different orders of magnitude were chosen

(5) $$\begin{array}{c} \beta_{i}=10^{i}\left[{\text{sec}}^{-1}\right]i=\bar{0,2}. \end{array}$$

With the relaxation function (3), the stress at time *t* + Δ*t* can be written as

(6) σ(t+Δt)=∫0t+ΔtG(t+Δt−τ)∂σ(ε)∂τdτ=∫0tG(t+Δt−τ)∂σ(ε)∂τdτ+∫tt+ΔtG(t+Δt−τ)∂σ(ε)∂τdτ.

After calculation of both terms of (6), the formula of stress at time *t* + Δ*t* can be written as

(7) σ(t+Δt)=G∞ε(t+Δt)+∑i=1e−βiΔt∫0tGie−βi(t−τ)∂σ(ε)∂τdτ +ε(t+Δt)−ε(t)Δt∑i=1(1−e−βiΔt)Giβi.

If the coefficients of the relaxation function (*G*_*i*_, *β*_*i*_) are known, the stress at time *t* + Δ*t* can be calculated based on the stress at time *t* and the strain at both time steps *t* and *t* + Δ*t*. The sum of squared errors (SSE) between the numerically calculated shear stress and the test shear stress at about 200 equidistant points on the logarithmic time scale was considered as the objective function. While the values of *β*_*i*_ were assumed (see (5)), the shear coefficients *G*_*∞*_ and *G*_*i*_ (4 optimization variables) were determined by minimizing the SSE using a quasi-Newton algorithm implemented in the Excel Solver package (Microsoft, Redmond, WA). The parameters of the LV model were identified under two conditions: one which neglects the loading curve assuming a perfect step shear loading [11] and the other which considers the whole loading curve [9]. These LV models are referred in this study as the analytical-based model without ramp and with ramp, respectively.

### 2.3. Material Identification: Finite Element Approach

**Figure 1:**
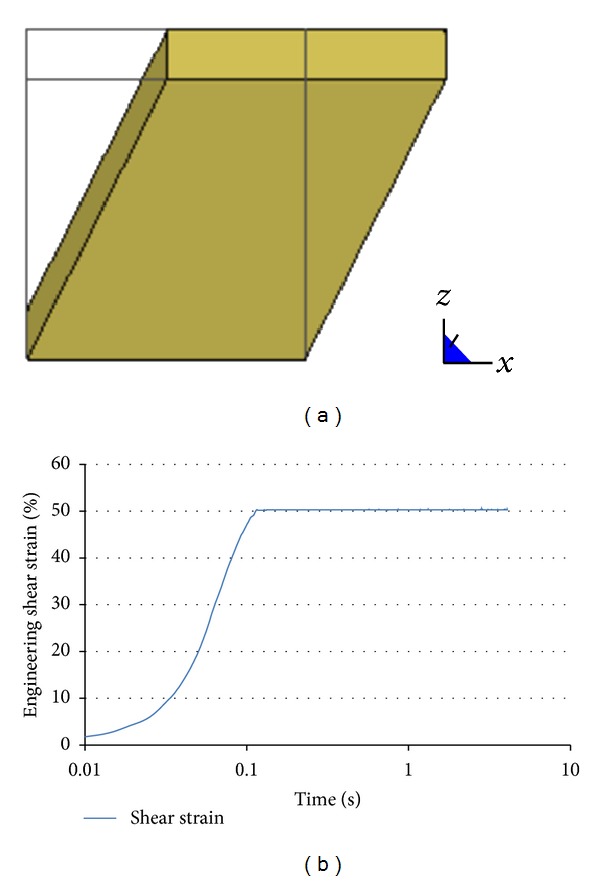
(a) Single element cube used to verify the FE material model against analytical solution. (b) Typical time history of shear strain applied.

An LV material model (Mat. 76, LS-Dyna Manual) was identified in LS-Dyna material library (LSTC, Livermore, CA) and employed in this study. Rate effects are taken into account in this LV material model through Boltzmann integrals (4) for both changes in shape and in volume. Therefore, the model allows for inputs relaxation functions represented using Prony series for both shear and bulk moduli. A relaxation function with four terms similar to that used in the analytical formulation ((3) and (5)) was employed, while the bulk modulus was assumed constant (rate independent) with a value of 2.1 GPa [16] similar to that of water. To check if the analytical formulation and the FE LV formulation are similar, a simple sanity check was performed (Figure 1(a)). A shear displacement, based on a typical shear input used in testing (Figure 1(b)), was applied to one face of a single element cube with dimensions 1 × 1 × 1, while the opposite side was fixed. The time history of shear stress was recorded within the element and compared with the shear stress calculated by the analytical formulation. 

**Figure 2:**
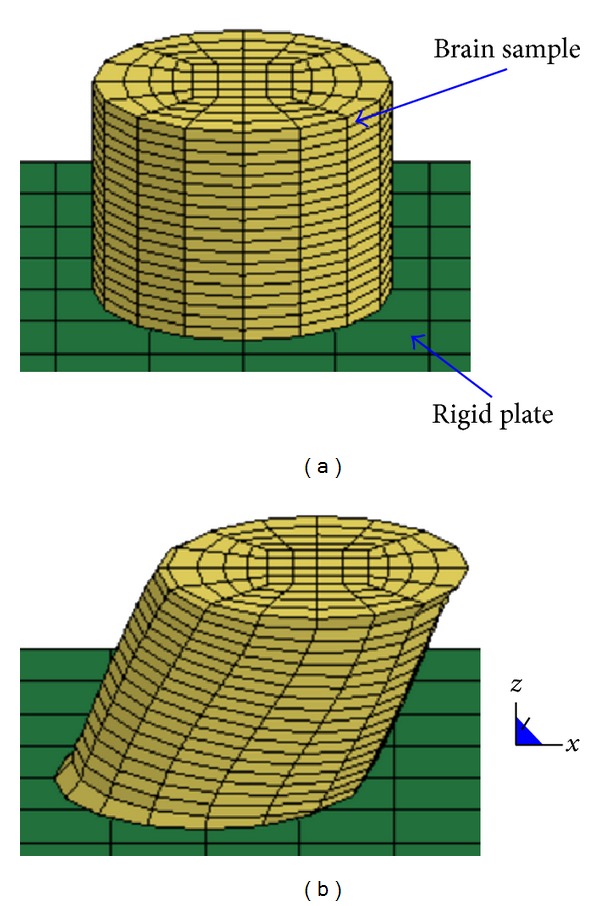
The FE model of brain samples: (a) undeformed state, (b) deformed state (50% engineering shear strain).

A parametric mesh of the cylindrical brain sample was developed in TrueGrid (XYZ Scientific Applications, Inc., Livermore, CA) and all seven brain specimens were modeled. While the model nodes of the downward face were fully constrained to the rigid plate, the model nodes of the upward face were constrained only in the *z* and *y* directions. Displacement in the *x* direction was prescribed based on the displacement time history measured during testing. The time histories of shear force were calculated as the sum of nodal forces of the downward face along the *x* direction (Figure 2). 

**Figure 3:**
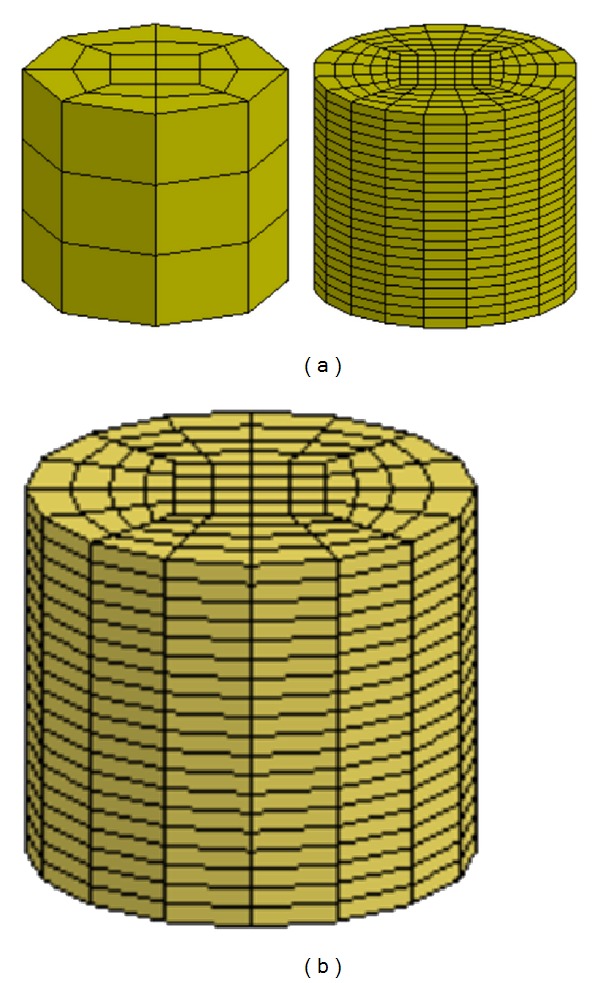
The specimen-specific brain model. (a) Intermediate FE models, (b) final FE model (1,520 elements).

A convergence study was performed by varying the mesh density of a specimen-specific model in a range from 176 to 3,209 elements (Figure 3(a)). It was observed that the force-time history predicted by a model (1,520 elements—Figure 2(b)) with hexahedral elements having the lengths in a range from 0.8 to 3 mm does not exceed 0.05% the corresponding data of the finest model. Therefore, a similar mesh density was used in all specimen-specific models.

The shear force-time history predicted by the FE model using the analytical-based models showed lower peak stresses during the loading ramp in all test simulations. Therefore, a FE-based material identification was performed to fit the test data. As in the material identification of analytical model, the shear moduli (*G*_*∞*_, *G*_*i*_) were considered as input variables, and the SSE of shear forces between the test data and corresponding model data was defined as the objective function to be minimized. The successive response-surface methodology (SRSM), an iterative statistical optimization method implemented in LS-Opt ver. 4.2 (LSTC, Livermore, CA), was employed to find a parameters set which minimize the objective function. A D-optimal design was used to search the test points around the optimum point determined after each iteration [5, 17] and a quadratic response surface was fitted through the values of the objective function calculated from FE simulation. The optimum point obtained using analytical model was considered as initial guess, and the optimization process was stopped after 5 iterations. Then, FE simulations using specimen-specific models, corresponding to each brain sample with three material parameter sets obtained by analytical-based optimization with and without ramp loading and by FE-based optimization, were performed and compared to test data.

While both analytical solutions showed a softer response, a sensitivity study was performed to see the effect of the specimen height and cross-sectional shape. The specimen height was varied from current height until a reduced height (0.7 h) was reached while keeping the same cross-sectional area. The displacement input was scaled in order to apply the same maximum shear strain (50%) and the analytical-based material model optimized with ramp was assigned to the sample model. The same parametric study with respect to specimen height was repeated using cubic specimens with the same cross-sectional area as cylindrical specimens. In all FE simulations the shear force was calculated and compared to test data.

## 3. Results and Discussion

**Figure 4:**
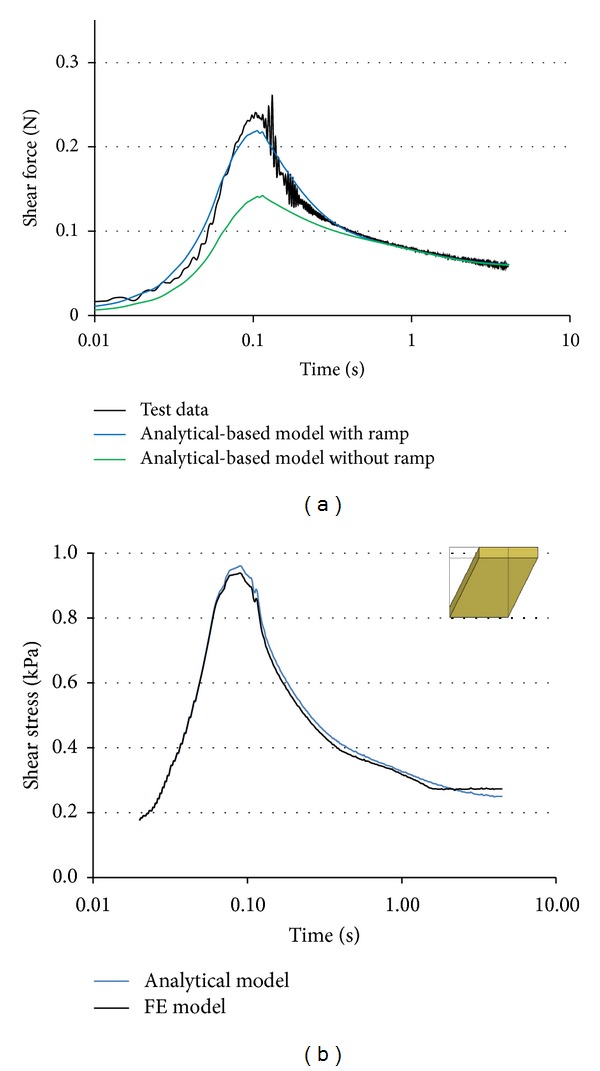
(a) Analytical (1D) model. Comparison between the test data and analytical-based optimized models with and without strain ramp in a typical test. (b) Cube (3D) model. Comparison between the shear stress responses of cube model using analytical-based model with ramp FE solution versus analytical solution.

The time histories of the shear force (unfiltered) show little noise during loading and relaxation phases, but inherent oscillatory mechanical noise occurred at the beginning and at the end of the ramp phase (Figure 4(a)). These oscillations, caused by inertial effects, were eliminated in the analytical parameter identification by minimizing the SSE of the model fit. The LV constitutive model that was optimized considering the loading ramp shows a good fit to the test data (Figure 4(a)). As expected, the model optimized by neglecting the loading ramp was able to characterize the relaxation response but underpredicted the peak stresses during the loading phase (Figure 4(a)). 

For the same set of LV material coefficients identified based on the analytical approach, the shear responses calculated using FE cube and using the analytical formulation has been close to each other (Figure 4(b)). A slightly higher peak was predicted by the analytical formulation (~1-2%), which assumed the brain as a perfect incompressible material, compared to the FE formulation that employed a nearly incompressible assumption (LS-Dyna Mat. 76). 

**Figure 5:**
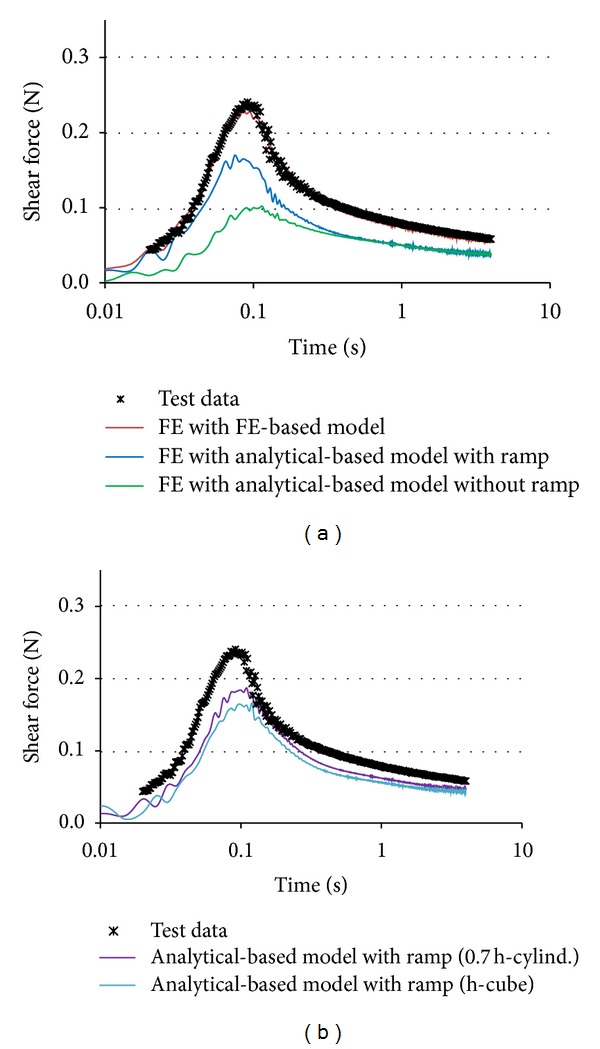
FE (3-D) sample model. Comparison between the test data and (a) FE predictions of the FE-based optimized model and analytical-based models with and without strain ramp in a typical test, (b) FE prediction of the analytical-based models with cubic sample (original height—h) and cylindrical sample (reduced height—0.7 h).

The shear force-time histories of the brain sample model with material coefficients obtained by the FE-based optimization matched the test data well (Figure 5). However, the shear force response of the same sample model with analytical-based material model with the strain ramp showed lower peak forces and relaxation response than the test data (Figure 5(a)). The cubic shape of sample showed a similar response like the cylindrical samples in terms of peak force with a delay during the loading phase. The reduced height of the sample increases the force peak, but the response is still softer than the test data (Figure 5(b)). 

**Figure 6:**
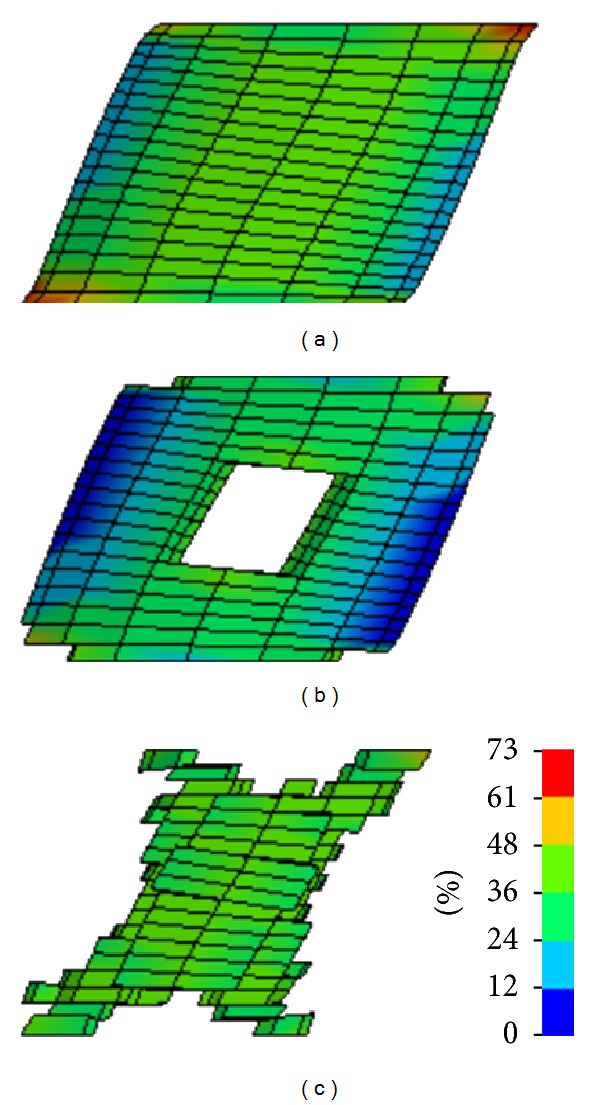
The shear strain distribution within the cylindrical sample at maximum displacement: (a) whole model (1520 elements), (b) elements with shear strain less than 45% (773 elements), and (c) elements with shear strain between 45% and 55% (665 elements).

**Figure 7:**
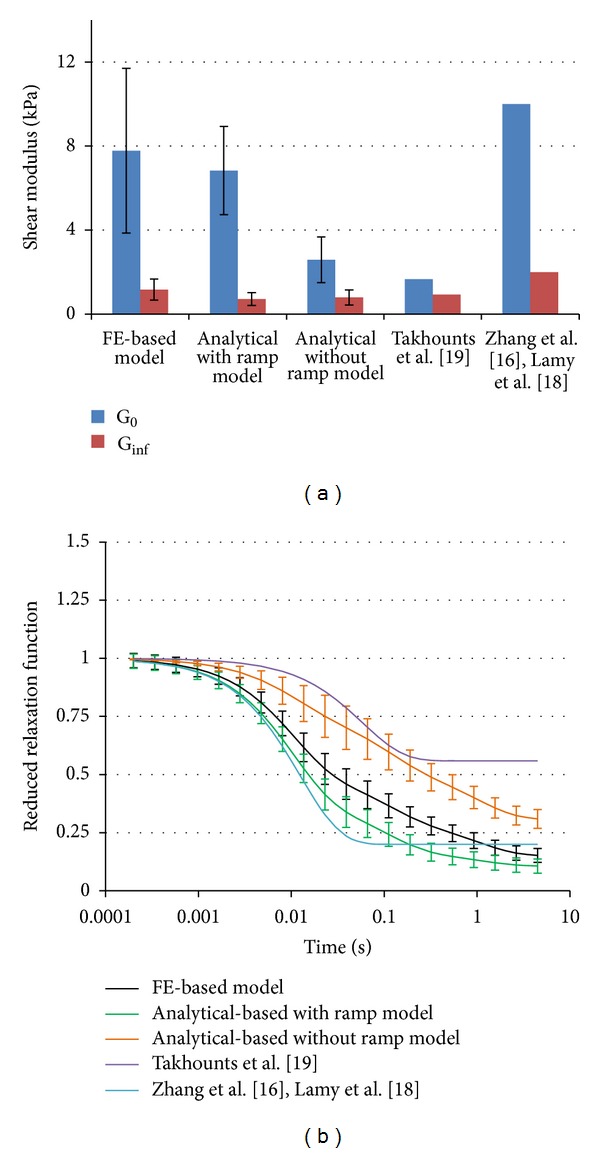
(a) The average instantaneous (*G*_0_) and the long term (*G*_*∞*_) shear moduli. Comparison between the material models obtained in this study and the literature models. (b) The average shear relaxation functions.

Different performances of the same parameter set in 1D and 3D models could be explained by the violation of the assumption of uniform distributed shear strain within the cylindrical sample. While a constant shear strain is realized in the cube (1D) model in the cylindrical sample, in the (3D) model the shear strain distribution is more complex (Figure 6). For example, at the maximum shear displacement, 50.9% (773 elements, Figure 6(b)) of the total number of elements (1,520 elements) have the shear strains under 45%, and only 43.8% (665 elements) are in a range from 45% to 50% shear strain, which is close to the assumed strain (50%). Therefore, the lower shear force predicted by the analytical model can be explained by the high percentage of elements with shear strains at lower levels than assumed by the analytical approach. The sample FE model with material coefficients obtained by analytical-based optimization approach without the ramp input shows the softest response of all models (Figure 5) This behavior is caused by the lower values of the instantaneous shear moduli corresponding to this model relative to other models that included the loading region in the identification process (Figure 7(a)).

Both average instantaneous and long term shear moduli of the FE-based optimized model show higher values than the corresponding values of the analytical models, but there are in the range of material data used in current FE brain models [16, 18, 19] (see Figure 7(a)). In addition, the average reduced relaxation function of the FE-based material model appears to be closer to the corresponding average function of the analytical-based model optimized with ramp. The reduced relaxation functions used in current FE brain models used only one exponential term and are above [19] or under [16, 18] the average functions reported in this study (see Figure 7(b)). 

The results of the current study suggest that the shape of tested specimens has an important influence on the shear coefficient values identified by an analytical approach. It should be also mentioned that other test conditions, such as temperature, anisotropy, and precompression of sample, may influence the identified material properties of the brain [20]. Reducing the height of specimens improves the performance of analytical-based optimized models, but no improvements were observed in cubic specimens. While the FE-based identification method requires a higher computational cost, it showed the best performance [21] and it is recommended to be applied in future. To increase the biofidelity of brain models, it is recommended to measure the normal (tensile) force in future tests in addition to the shear force and use it during the optimization process. Furthermore, advanced material models (e.g., nonlinear models) should be implemented in current FE software in order to better replicate the complex behavior of the brain in the human FE models. 

The reduced number of tests analyzed and their relatively noisy time histories of force limit using the current data for defining a reliable material model of human brain. However, in the future, we believe the observations reported in this study will be useful for defining better test protocols and more accurate test data analyses in an effort to develop more biofidelic brain FE models. 

## 4. Conclusions

The material properties of the human brain under large shear deformation were investigated in this study based on test data recorded on cylindrical specimens. The models optimized using only the relaxation curve predict much lower stresses during loading mainly due to low values of their instantaneous shear moduli. In addition, the material models optimized using an analytical model which assumes a uniform strain distribution predict lower forces in finite element simulations. Finite element optimization appears to be a promising tool for the identification of brain material properties by considering the entire loading time histories and nonuniform strain distribution within the sample.
